# Somatic Maintenance Resources in the Honeybee Worker Fat Body Are Distributed to Withstand the Most Life-Threatening Challenges at Each Life Stage

**DOI:** 10.1371/journal.pone.0069870

**Published:** 2013-08-05

**Authors:** Siri-Christine Seehuus, Simon Taylor, Kjell Petersen, Randi M. Aamodt

**Affiliations:** 1 Department of Aquaculture and Animal Sciences, Norwegian University of Life Sciences, Aas, Norway; 2 Queen Maud University College, Early Childhood Education, Trondheim, Norway; 3 Centre for Integrative Genetics (CIGENE), Norwegian University of Life Sciences, Aas, Norway; 4 Computational Biology Unit, Uni Computing, Uni Research AS, Bergen, Norway; Leibniz Institute for Age Research – Fritz Lipmann Institute (FLI), Germany

## Abstract

In a global transcriptome analysis of three natural and three manipulated honeybee worker phenotypes at different ages, we have investigated the distribution of investment in somatic maintenance of the fat body. Gene expression is modulated so that the bees are able to resist the most life-threatening challenges at the actual life stage. Different modes of maintenance and repair are regulated, apparently to meet the environmental challenges most detrimental to survival and reproductive potential for the hive. We observed a broad down-regulation of genomic and cellular maintenance in the short-lived foragers and nurse bees compared to the long-lived winter bees. Our results show that survival and reproduction of the entire hive is given priority over the individual bees, hence supporting the idea of the honeybee society as a superorganism. Our results also fit the disposable soma theory of aging.

## Introduction

In the insect world a broad array of different forms and degrees of social organisation can be found. In the more highly developed forms, social insect colonies are so tightly integrated that they have been suggested to function as a single organism, a superorganism [1], [2], [3]. Honeybees are eusocial insects where the fundamental components that underlie superorganismal order are the female forms [4]. Like sterile somatic cells, the workers differentiate and communicate to produce coordinated patterns of growth, homeostasis, provisioning and defence before death [4]. In the *Apis mellifera* subspecies in Old World temperate climates, workers spend the first weeks performing in-hive activities like brood care (nurse bees), and switch at age 3–4 weeks to foraging tasks (foragers) [5], [6]. Workers emerging in autumn develop into so–called *diutinus* [7] or winter bees that survive for 8–10 months [8], [9], [10]. The following spring, winter bees will begin either nursing or foraging, and simultaneously start to age [7].

We have performed a global transcriptome analysis of the fat body of three naturally occurring honeybee worker phenotypes, in addition to three manipulated worker types. The fat body is the insect equivalent to white adipose tissue or liver [11]. We observed a broad down-regulation of the somatic maintenance machinery in the foragers. In nursing bees, body maintenance is extensively up-regulated at the external level, i.e. the cuticula. In the long lived winter bees however, but not in the other phenotypes, a broad array of maintenance and repair functions of cells, proteins and nucleic acids took place. Different modes of maintenance and repair thus seem to be expressed to meet the environmental challenges most detrimental to survival and reproductive potential at the actual life stage. If a clear pattern of resource allocation between reproduction and repair and between different modes of maintenance and repair could be observed on a colony level, in would support the theory of the honeybee society being a superorganism, since such a pattern would indicate that the whole society must be under selection and not only its individual parts. We therefore consider that these results support the concept of the honeybee society as a superorganism, and also give substantial support to the disposable soma theory of aging [12] at this level of biological organisation. If the disposable soma theory is correct, an ageing programme would be expected to spare crucial functions but to down-tune others to a level just necessary to sustain functionality for the remains of an estimated life span under the actual environmental risks and circumstances [13]. This theory states that aging results from the body's need to budget the amount of energy available, resulting in imperfect maintenance of the soma.

## Materials and Methods

### Nurse bees and foragers

Honeybees were paint marked on emergence and introduced to production hives in the apiary of the Norwegian University of Life Sciences (Aas, Norway). After eight days painted bees with head and thorax in cells containing larvae were gathered as nurse bees. Foragers were marked at the hive entrance in a second colour. When most of the marked bees were verified foraging, the double marked returning foragers were gathered at the hive entrance. After removing the intestines the bees were rapidly frozen on dry ice and transferred to −80°C for storage until further processing.

### Reversion

Approximately 6000 bees were marked on emergence and introduced into a six frame host colony containing a mated queen, unmarked bees of various ages, brood and food. The host colony had an empty hive unit on top. Entrance counts of marked bees returning from foraging were performed four times daily. When the number was declining, the hive was manipulated to separate marked foraging bees from marked foragers still engaged in hive activities. At the high of foraging activity, the host colony was removed from its original site. The queen was replaced in a new host colony with three frames of open brood and two frames of honey and pollen, but devoid of worker bees. The new hive box was placed at the original site of the colony with the original flight entryway. The old host colony was placed on top with a divider between the two hive boxes. The top box got a new entranceway 180 degrees from the original one. Experienced foragers returned to the new hive box. Four and eight days after reversal nursing foragers were collected and frozen as described.

### RNA interference and methoprene treatment

Maurizio hives were established by caging the queen, removing all open brood from the host colonies and giving an excess of food. After most of the sealed brood had emerged RNA interference knockdowns were established as described [14]. Newly emerged workers were injected with 1.5 μl of dsRNA (5 μg/μl). The dsRNA was synthesised by RiboMax Express Large Scale RNA production system (#P1320, Promega) with DNA template from *vitellogenin* (GenBank accession no. AJ517411) clone *Ap4a5* (kind gift of Dr. Zilá Luz Paulino Simões). The injected bees were marked with a spot of paint and introduced in equal amounts into two Maurizio hives. After approximately one week marked bees were gathered and frozen as described. Simultaneously, adult workers from two Maurizio hives were gathered and treated with methoprene by dripping 5 μl (40 μg/μl) of the solution on the thorax and abdomen of the bee (approximately 200 μg per bee). The workers were allowed to dry before being marked and reintroduced into their original colonies. After 48 hours the workers were collected and frozen as described.

### Winter bees (diutinus bees)

Winter bees were collected from the winter cluster of normal production hives in the middle of the Norwegian winter season (mid December). The bees were immediately frozen as described.

### RNA extraction and transcriptome sequencing

Nurse bees, foragers, winter bees, *Vg*Knocdowns, methoprene treated bees and reversion bees were all subject to RNA extraction. The extraction was preformed according to the RNAeasy Mini Kit (#74106, Qiagen) procedure with slight modifications. In brief; the frozen bees were disrupted and homogenized with Precellys 24. The lysate were treated according to protocol. After the washing steps the columns were incubated with DNase mix (20 μl DNase I stock solution 60 μl RDD buffer as provided by the kit) for 15 minutes at room temperature, before further washing and elution of the RNA as according to kit protocol. The RNA was frozen at −80°C in 20 μl aliquots. From each sample 2 μl was tested for quality and concentration by the Agilent RNA 6000 Nano Assay (Bioanalyzer) and by Nanodrop. Only samples that showed a curve consistent with no degradation of the material and with a total concentration above 7.5 μg were considered viable for sequencing. We chose to send five individual samples of nurses, foragers, *vg*knockdowns, methoprene treated bees, three samples of reversion bees sampled day 4 after reversion and two samples day 8 after reversion. The winter bees were pooled in samples of four and five individuals. Two samples of four individuals and two samples of five individuals were sent to Uppsala Genome Centre, Rudbeck laboratory for whole transcriptome sequencing.

### Mapping of reads and annotation of resulting protein matches

The colour-space reads for each individual were mapped separately to the nucleotide reference (Gnomon_mRNA.fsa NCBI build 4.1, August 2006) using version 1.2.1 of the SOLiD^TM^ WT Analysis Pipeline. The “pbs” option was set to utilise the batch queuing system and all alignments were done on a 12 core Ubuntu Linux server with 64GB RAM. The mismatch count was limited to two and the minimum mapping score was set to 24. The remaining options were left at their default settings.

The resulting GFF files were binned into a ‘hit list’ of the number of read alignments per mRNA contig. These contigs were then extracted from the reference and formed the basis of the protein and gene search. Proteins contained in each high-hitting contig were found from a blastx search (filtered with an expectation value of 1e–20) against a local copy of the NCBI protein database. Any with a percentage match outside of the 99–100% range were rejected from further analysis, leaving a high quality list of approximately 15000 matches per individual. The list was further reduced by filtering out contigs that mapped multiple times to the same protein by selecting those with the lowest mismatch count, typically 0 or 1. Unique proteins that mapped multiple times to the same reference contig were retained for analysis. The resources required to complete all the blasts in a reasonable time were somewhat higher than the alignment hardware, so the Norwegian High-Performance computing cluster (titan) was utilised.

The FASTA header for each protein in the database used above conveniently contains an accession number which was further used to discover its parent gene. This was done using the NCBI Entrez E-utils web interfaces: *esearch* and *esummary*. The *esearch* tool was called first using the “db = gene” parameter with the accession number used as the search term. The returned gene id was then passed to the *esummary* tool which provided detailed gene information.

Final output of all the steps was one file for each bee containing the highest hitting contigs with associated proteins and gene details. The resulting file consisted of 28 samples divided in the following behavioural groups: nurses, foragers, winter bee pools, *vg*knockdowns, methoprene treated bees and reversion bees; 8640 transcripts across all groups.

### Quantitative transcript expression analysis

The read counts per contig for the 28 samples were normalized with quantile normalization [15] as implemented in J-Express 2011 (jexpress.bioinfo.no [16]), and missing values were replaced with the count number 1. This pre-processing facilitates pairwise Rank Product [17] comparisons of the different honeybee sample groups. These normalized read based expression profiles were further mean centred before Hierarchical clustering was performed (Average Weighted Linkage WPGMA, Euclidean distance measure).

To assess global relationships between samples, we performed hierarchical clustering of samples over the 8640 transcripts in J-Express using Pearson correlation as distance measure and average weighted linkage (WPGMA). This analysis produces a heat map of Pearson correlation values between all samples in addition to the dendrogram with internal relationships of the samples. The normalized and log2 transformed dataset was mean normalized before the clustering, centring the transcript profiles around 0 on the log2 scale. As a second view on the global relationships between samples, we performed a Correspondence Analysis in J-Express on the normalized and log2 transformed dataset.

### Gene Ontology annotation

Every single gene id from the blast was mapped for the corresponding entity through Flybase (http://flybase.org), NCBI homologene (http://www.ncbi.nlm.nih.gov/homologene), Uniprot (http://www.uniprot.org), or NCBI nucleotide blast similarity search (http://www.ncbi.nlm.nih.gov/nuccore). We used the resulting *Drosophila* Gene Ontology association file dated May 3^rd^ 2011, and mapped the id's to Gene Ontology terms in the obo file dated Dec 3^rd^ 2010.

### Functional subsets of genes for clustering analysis

From the initial GO annotation, extensive literature mining and Kegg pathway annotations (http://www.genome.jp/kegg/pathway.html) we created subsets of genes relevant for ageing for further analysis. To find genes with similar expression patterns within the functional categories we performed a focussed hierarchical clustering on each subset in J-Express.

### Verification of transcriptome data, QPCR

To verify the transcriptome data a QPCR was performed using 2 bees from each group: i) nurses, ii) foragers, iii) methoprene treated bees, iv) *vg*knockdown bees, v) 4 day old reversion bees, and vi) winter bees. The cDNA synthesis was performed according to kit specifications, SuperScript Vilo, cDNA synthesis Kit (#11754-050, Invitrogen), using 1.5 μg RNA as input. The primers were designed using NCBI blastn and Primer3 ((http://www.bioinformatics.nl/cgi-bin/primer3plus/primer3plus.cgi), (Invitrogen)) ( Figure S1, Table S4, primers and plate set up). The final QPCR was performed with Fast SYBRGreen Master Mix (#4385616, Invitrogen). To be sure that we had a stable expression of our reference gene, three genes were tested initially: i) NCBI gene id 551343, GB15053, *rpt1*; ii) NCBI gene id 406099, GB10903, *rpL32*; and iii) NCBI gene id 552649, GB13210, *ef1beta*. Of these three, *rpL32*, was most stable in our material and the two others were discarded in the final setup. Nine genes were tested for relative expression in our final setup; i) NCBI gene id 412027, GB15777, *pr-set7*; ii) NCBI gene id 727091, GB13873; *foxo*; iii) NCBI gene id 725827, *igf1rl*; iv) NCBI gene id 406088, GB13999, *vg*; v) NCBI gene id 726182, GB15055, *vhld*; vi) NCBI gene id 406144, GB18323, *abaecin*; vii) NCBI gene id 494509, GB15139, *malvolio*; ix) NCBI gene id 412846, GB17426, *Sirt6*; x) NCBI gene id 409532, GB12793, *Sirt2*.

## Results

The resulting file after blasting and cut-off consisted of 8640 transcripts over 28 individual samples. We have chosen to present the results according to the behavioural groups in the dataset rather than as individual samples. The SOLiD whole transcriptome dataset vas validated by qPCR and there was a very good correlation between the results in all comparisons (see table 1).

**Table 1:** qPCR validation of SOLiD whole transcriptome data.

expressed genes	Nurses vs. *vg*knockdowns		Nurses vs. Methoprene bees		Nurses vs. Reversion bees		Nurses vs. Foragers		Nurses vs. Winter bee pools	
	exp. data	qPCR	exp. data	qPCR	exp. data	qPCR	exp. data	qPCR	exp. data	qPCR
**Abaecin**	20,66	9,36	1,89	4,5	11,05	6,91	7,89	16,53	6,34	5,84
**FOXO**	1,18	2,62	−1,21	2,1	1,22	3,3	1,14	2,54	1,2	1,54
**IGF1R**	1,21	2,43	1,39	2,23	1,29	20	−1,24	2,59	−3,48	1,69
**Malvolio**	14,49	21,01	6,24	13,08	7,14	25,53	6,2	21,23	2,91	2,97
**Pr-set7**	1,25	2,24	1,13	3,5	1,2	2,44	1,04	1,97	1,89	1,94
**Sirt2**	−1,28	1,02	−1,5	−1,68	−1,57	1,46	−1,26	1,83	1,1	−2,66
**Sirt6**	−1,46	1,24	−1,43	1,19	−1,65	1,5	−1,52	1,84	−1,24	1,44
**vg**	−5,8	−278,91	−3,74	−3,84	−12,81	−13,4	−24,5	−14,72	−4,84	−6,28
**vhdl**	−50,44	−23,36	−48,13	−26,54	−112,43	−42,68	−36,54	−10,77	−4,7	−12,93
**Correlation**	0,180185323		0,959251623		0,879144028		0,843930067		0,757516225	

There was a very good correlation between the experimental data and the qPCR in all comparisons apart from nurse bees versus *vg*knockdowns. Omitting this data point gave a correlation of 0.93435774 for the rest of the genes in this comparison.

### Global analysis of samples

**Figure 1:**
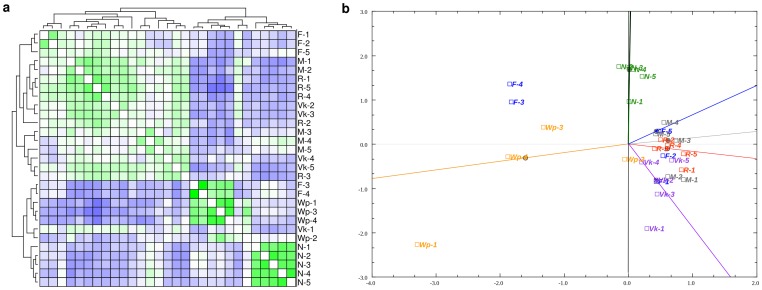
Global relationships between samples. a) Hierarchical Clustering of samples over all 8640 transcripts, using Pearson correlation and average linkage (WPGMA), range dark blue −0.75 to dark green 0.75. b) Correspondence analysis plot of samples only. Abbreviations: (F1-5) individual forager samples, (N1-5) individual nurse bee samples, (Wp1-4) individual winter bee pooled samples, (Vk1-5) individual *vg*knockdown samples, (M1-5) individual samples of methoprene treated bees, (R1, R2, R3) individual samples of reversion bees day 4 after reversion and (R4, R5) day 8 after reversion. (FC) fold change, (up N) up-regulated nurses, (up F) up-regulated foragers, (up Wp) up-regulated winter bee pools), (down N) down-regulated nurses, (down F) down-regulated foragers and (down Wp) down-regulated winter bee pools.

The analysis (CA) and hierarchical clustering of samples (figure 1, a and b) revealed a clear difference between the naturally occurring worker phenotypes; nurses (N1, N2, N3, N4, N5), foragers (F1, F2, F3, F4, F5) and winter bee pools (Wp1, Wp2, Wp3, Wp4). Whereas the manipulated groups, *vg*knockdowns (Vk1, Vk2, Vk3, Vk4, Vk5), methoprene treated bees (M1, M2, M3, M4, M5) and reversion bees (R1, R2, R3, R4) clustered with the foragers (figure 1 a). The right panel shows the sample plot from the Correspondence Analysis (figure 1 b). The projection of the data along the first axis (x) explains 7.57% of the variance, while the second axis (y) explains 6.15% of the total variance in the data. All five nurse bees clustered together in both analyses, whereas the winter bee pools and the foragers were less consistent. The largest differences were in the foragers where two of the samples were different from the other three (figure 1, b; F1, F2 and F5 versus F3 and F4). The foragers were sampled at the flight entrance and consisted of individuals with various days of foraging experience. Both methoprene treated bees (M1-5), reversion bees (R1-5) and *vg*knockdowns (Vk1-5) tended to cluster together with three of the forager (F1, F2 and F5) (figure 1, b).

### Global analysis of genes

**Figure 2:**
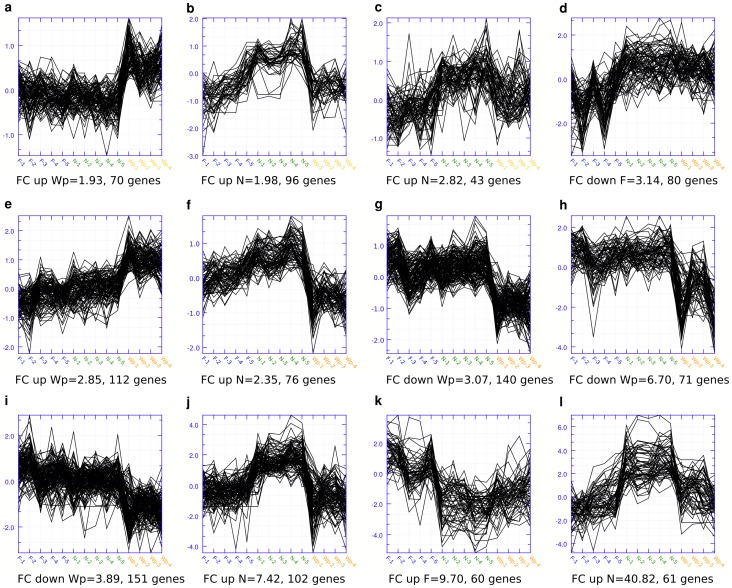
The resulting extraction of 12 clusters from the unsupervised hierarchical clustering analysis of all 8640 transcripts. Only foragers, nurses and the winter bee pools were included in the analysis. The x axis denotes the fold change and the y axis the individual bees within each worker group. Two clusters were up-regulated in winter bee pools (a, e), whereas four clusters were down-regulated in this worker phenotype (f, g, h and i). Four clusters were up-regulated in nurse bees (b, c, j, and l); one cluster was up-regulated in foragers (k) and one down-regulated (d). Abbreviations: (F1-5) foragers, (N1-5) nurses bee, (Wp1-5) winter bee pools, (FC) fold change.

In an initial unsupervised hierarchical cluster analysis of all genes in the dataset of all worker phenotypes the naturally occurring worker phenotype sample groups showed strong consistent trends over the replicates in the resulting heat maps. The manipulated worker phenotypes did not show unique trends in this analysis with the natural groups (data not shown). Clustering the genes of the data set with the three naturally occurring worker phenotypes only, showed clear differences in the resulting heat map. From this heat map we chose the 12 clusters with distinct and consistent differences in expression levels between the worker groups (figure 2, see also Table S1). Overall, six of the 12 clusters were either up-regulated or down-regulated in winter bees (Wp) compared to the two other groups. Four were up-regulated in nurses (N) and two were up- and down-regulated in foragers (F). We chose to describe expressed genes within the functional categories with special relevance to aging.

#### Clusters up-regulated in winter bees compared to nurses and foragers

*Cluster 1* (figure 2, a; fold change 1.93, 70 genes): In this cluster we find seven genes annotated as cell growth and development and three as determination of adult life span, amongst those *target of rapamycin* (*tor*, GB11213). In the DNA binding group we find three DNA repair enzymes, one in the mismatch repair (MMR) pathway, *mutL homolog 1, colon cancer, nonpolyposis type 2 (E. coli)* (*mlh1*, GB17468) and one in the nucleotide excision repair (NER) pathway, *xeroderma pigmentosum group A-like* (*xpac*, GB14782). Eleven expressed genes are involved in transcription and six in translation, among them *RNA polymerase 1 subunit* (*rpl1*, GB12611) and *eukaryotic translation initiation factor 3 subunit L* (GB11201) and *transcription factor IIE alpha* (*tfIIealpha*, GB17516).

*Cluster 5* (figure 2, e; fold change 2.85, 112 genes): In this cluster we find eleven expressed genes annotated as cell growth and development, three chaperone proteins, one chromosome maintenance gene and seven genes annotated as DNA binding, metabolism, replication and repair, most of which are involved in DNA replication. We also find the *histone ribonuclease H2 subunit A* (GB14057), *similar to CG5585-PA* (GB17388), which is a histone methyltransferase, and *similar to CG32165-PA* (*importin*, GB11019) in this group. The main group in this cluster is 25 nuclear ribosomal proteins, showing high transcription activity. Eight genes annotated as transcription belong in this cluster, as well as eight genes involved in translation.

#### Clusters down-regulated in winter bees compared to nurse and foragers

*Cluster 6* (figure 2, f; fold change 2.35; 76 genes): In this cluster there were five expressed genes in the annotation group cell growth and development. The genes were involved in eye and wing function as well as epidermal growth, presumably due to being of less importance to winter bees. The two chaperone proteins in this cluster are detoxification enzymes specific for mitochondria, *superoxide dismutase 2, mitochondrial* (*sod2*, GB14346) and *cytochrome c oxidase subunit 6A1, mitochondrial* (GB10253). The cluster also had 10 genes involved in energy gaining pathways, reflecting less energy requirement of the winter bees that neither fly nor feed larvae. Notably, the immune genes down-regulated by winter bees did not include antibacterial peptides.

*Cluster 7* (figure 2, g; fold change 3.07, 140 genes): In the annotation group autophagy, apoptosis and cell death, four genes were down-regulated, which implies that these processes are more important in foragers and nurses than in winter bees. Cell growth and development make a large group of 15 expressed genes and the term energy-gaining processes included 10 genes. In the immune defence term none of the genes were antimicrobial peptides. The genes in the behaviour, sensory, learning and memory term reflect the profile of the non-nursing, non-flying winter bees, including for instance *ecdysteroid-regulated gene E74* (*e74*, GB10759), involved in neural function and reproductive behaviour, several genes involved in phototransduction as well as *similar to Hypoxia-inducible factor 1 alpha (HIF-1 alpha) (HIF1 alpha) (ARNT-interacting protein)* (GB16786), which is involved in protection against stress caused by low oxygen levels (hypoxia).

*Cluster 8* (figure 2, h; fold change 6.70; 71 genes): The two most interesting annotation groups in this cluster were pathways and hormones and transcription. The former contained mostly genes involved in detection of stimuli, but also *hormone receptor-like in 46* (*hr46*, GB10650), which is a putative ecdysteroid-response gene, genetically linked to determination of ovary size in workers. The latter annotation group included for instance *jim protein* (*jim*, GB11661) involved in regulation of chromatin silencing and *hairy* (*h*, GB14857) which is involved in response to hypoxia.

*Cluster 9* (figure 2, i; fold change 3.89, 151 genes): Most of the expressed genes in the behaviour, sensory, learning and memory annotation group are involved with aggressive behaviour, walking behaviour or phototransduction. We found *SNF1A/AMP-activated protein kinase* (*snf1a*, GB15108) and *JNK MAP kinase basket* (*bsk*, GB16401) in pathways and behaviour, and s*imilar to rab3-GEF CG5627-PA* (LOC552179) in cell growth and development. These genes have been linked with response to starvation and nutrient levels in other species.

#### Clusters up-regulated in nurse bees compared to winter bees and foragers

*Cluster 2* (figure 2, b; fold change 1.98, 96 genes): In this cluster we found nine expressed genes involved in energy gaining processes as well as *isocitrate dehydrogenase* (*idh*, GB14517), involved in the TCA cycle as well as several genes involved in stress response.

*Cluster 3* (figure 2, c; fold change 2.82, 43 genes): This cluster showed expressed genes in nearly all annotation categories. The most striking were the number of genes involved in fatty acid beta oxidation and in several categories which are involved in response mechanisms to oxidative stress. *Cluster 10* (figure 2, j; fold change 7.42, 102 genes): This cluster showcased the strong investment in cuticular proteins, chitinase and chitin metabolic process with 15 expressed genes in this category, most of which are vital for maintaining cuticular structure. This cluster also contained *vitellogenin* (*vg*, GB13999) a very high density lipoprotein which is known to be highly up-regulated in nurses, as well as *juvenile hormone esterase* (*jhe*, GB15327), which may play a major role in the regulation of juvenile hormone titre (disrupting the JH titre).

*Cluster 12* (figure 2, l; fold change 40.82, 61 genes): This cluster also sported eight genes involved in maintenance of cuticular structure and most interestingly *larval-specific very high density lipoprotein* (*vhdl*, GB15055), *hexamerin 110* (*hex110*, GB14361), *hexamerin 70c* (*hex70c*, GB13613) which all are known haemolymph and storage proteins. Further, six of the expressed genes are involved in lipid metabolism and eight in pathways and hormones.

#### Clusters down-regulated in foragers compared to nurses and winter bees

*Cluster 4* (figure 2, d; fold change 3.14, 80 genes): In this cluster we found only two genes annotated in cell growth and development. One of these, *actin-related protein 8* (*arp8*, GB10541), is involved in chromatin remodelling. The category cuticular proteins, chitinase and chitin metabolic process had five expressed genes, most of which are involved in synthesis, and the nine immune defence genes were mostly cytochrome P450 homologs.

#### Clusters up-regulated in foragers compared to nurses and winter bees

*Cluster 11* (figure 2, k; fold change 9.70, 60 genes): The largest annotation groups in this cluster were immune defence, xenobiotic metabolism, and stress response with 10 expressed genes and pathways and hormones with eight expressed genes. Among the former were antimicrobial peptides like *hymenoptaecin* (GB17538) and *defensin 1* (*def1*, GB19392) as well as *beta-1, 3-glucan recognition protein 2* (*gnbp1-1*, GB19961). In the latter group were genes involved in negative regulation of epidermal growth factor-activated receptor activity like *kekkon-1* (*kek1*, GB17490). This cluster also contained *malvolio* (*mvl*, GB15139) which is involved in sensory perception of sweet taste and has been reported to increase expression levels in honeybee foragers.

### Rank Product test q = 10%, GO annotation

**Figure 3:**
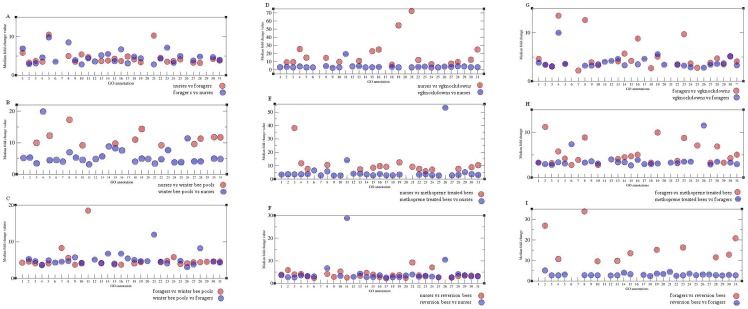
Median fold change value of the genes represented in each GO term within the comparisons. a) nurses versus foragers (red) and foragers versus nurses (blue), b) nurses versus winter bee pools (red) and winter bee pools versus nurses (blue), c) foragers versus winter bee pools (red) and winter bee pools versus foragers (blue), d) nurses versus *vg*knockdowns (red) and *vg*knockdowns versus nurses, e) nurses versus methoprene treated bees (red) and methoprene treated bees versus nurses (blue), f) nurses versus reversion bees (red) and reversion bees versus nurses (blue), g) foragers versus *vg*knockdowns (red) and *vg*knockdowns versus foragers (blue), h) foragers versus methoprene treated bees (red) and methoprene treated bees versus foragers (blue), i) foragers versus reversion bees (red) and reversion bees versus foragers (blue). The GO annotation terms are numbered 1–31: 1: aurophagy, apoptosis and cell death, 2: behaviour, sensory, learning and memory, 3: binding, 4: carbohydrate metabolic process, 5: cell growth and development, 6: chaperone, 7: chromosome maintenance, 8: cuticular proteins, chitinases and chitin metabolic process, 9: determination of adult life span, 10: DNA binding, metabolism, replication and repair, 11: energy, 12: histone, 13: immune defence, xenobiotic metabolism, stress response, 14: kinase, 15: lipid, phospholipid, fatty acid metabolism and fatty acid beta oxidation, 16: membrane, 17: mitochondrial proteins and mitochondrial ribosomal proteins, 18: motor proteins and muscle, 19: neurognesis, 20: nuclear ribosomal protein, 21: odorant binding proteins and receptors, 22: pathways and hormones, 23: protein metabolism, processing, stability, maintenance and repair, 24: receptor activity, 25: signalling, 26: TCA, 27: transcription, 28: transferase activity, 29: translation, 30: transport, 31: unknown function.

The Rank product test on the complete data set (table 2) was followed by a GO annotation on all expressed genes with a cut off of q = 10% (see table S2). The manipulated groups were compared to nurses and foragers since all the manipulations showcase the transition from nursing to foraging or the reverse process. The individuals within the manipulated groups varied somewhat in their expression (figure 1, a and b), still the comparison with a cut off value of q = 10% showed some interesting patterns. Only the genes with the highest fold change in expression will be described (figure 3 a – i), for a more minute description see table S2. To visualize the GO terms with the highest fold changes we extracted the median fold change value of each GO annotation term in each comparison as well as the minimum and maximum value within each term, see Tables S5–S13 in File S1and tables therein. The median fold change value of each term is depicted in figure 3.

**Table 2:** Number of differently expressed genes with a cut-off of q = 10% in all comparisons of worker phenotypes.

	Nurses	Foragers	Methoprene bees	*vg*knockdowns	Reversion bees	Winter bees
**Nurse**		259	68	71	359	58
**Forager**	240		154	169	28	303
**Methoprene**	270	124		71	74	362
**Vg knockdown**	253	78	57		70	273
**Reversion**	326	102	73	79		440
**Winter bee**	226	144	208	211	259	

#### Nurses versus foragers (figure 3 a)

In this comparison, the annotation terms with the largest number of expressed genes were binding, cuticular proteins, chitinase and chitin metabolic process, immune defence, xenobiotic metabolism, stress response, pathways and hormones and transport, apart from the genes that annotated as unknown function. The genes in the cuticular proteins, chitinases and chitin metabolic process were mostly involved with chitin metabolism or essential for maintenance of chitin structure; the *hypothetical LOC552685* (GB11550, fold change 191.85) may possibly also be a cuticular protein. Apart from these annotation groups, there were four genes in olfactory annotation group: *odorant binding protein 3* (*obp3*, GB19454, fold change 3.16), *odorant binding protein 13* (*obp13*, GB18363, fold change 13.31), *odorant binding protein 14* (*obp14*, NP_001035313.1, fold change 72.54) and *odorant binding protein 17* (*obp17*, GB11092, fold change 7.34). Interestingly, we observed a 3.96 fold change in the *similar to 5-hydroxytryptamine 1A receptor (5-HT-1A) (Serotonin receptor 1A) (5-HT1A)* (*5ht1a*, GB14262) and a 45.41 fold change in *similar to pericardin CG5700-PB* (GB19347); the former is possibly involved in social behaviour and the latter in maintenance of the heart. The haemolymph and storage proteins *vitellogenin* (*vg*, GB13999, fold change 12.93), *larval-specific very high density lipoprotein* (*vhdl*, GB15055, fold change 17.79), *hexamerin 110* (*hex110*, GB14361, fold change 25.01), *hexamerin 70c* (*hex70c*, GB13613, fold change 4.63) and *hexamerin 70b* (*hex70b*, GB10869, fold change 3.93) were all up-regulated in nurses compared to foragers.

#### Foragers versus nurses (figure 3a)

In the reverse comparison, most of the expressed genes in the behaviour, sensory, learning and memory category were involved in adult feeding behaviour and sensory perception of chemical stimuli and sweet taste. The foragers also up-regulate genes involved in the production of dopamine, like *tyrosine hydroxylase* (*tyhyd*, GB15303, fold change 5.59), an enzyme responsible for production of L-DOPA, and *similar to Aromatic-L-amino-acid decarboxylase (AADC) (DOPA decarboxylase) (DDC)* (GB14019, fold change 9.72), catalysing the rate-limiting step in the production of dopamine. Dopamine is involved in the regulation of motivation and reward in insects as well as in other species. The immune defence, xenobiotic metabolism and stress response category contained 31 genes; among these were several antimicrobial peptides, multiple cytochrome P450's and others involved in stress response. Also *hypothetical LOC408807* (*irp30*, GB10708, fold change 62.28, category unknown), a possible secreted immune protein, was highly up-regulated. In this comparison there was an up-regulation of genes encoding lipid catalysis, proteolysis and proteases, suggesting a decimation of lipid storage in the fat body of foragers.

#### Nurses versus winter bee pools (figure 3b)

In comparison to the winter bee pools, nurses up-regulated genes involved in environmental information processing, phototransduction and *BM-40-SPARC protein* (*bm-40-sparc*, GB11432, fold change 10.01), which is suggested to be involved in regulation of circadian rhythm. In the category cuticular proteins, chitinase and chitin metabolic process there were 15 genes which mostly are essential for cuticula structure, including the possible cuticular protein, *hypothetical LOC552685* (GB11550, fold change 408.65) in the unknown function category. Also notable is the haemolymph and storage protein, *hexamerin 110* (*hex110*, GB14361, fold change 54.36).

#### Winter bee pools versus nurses (figure 3b)

In the reverse comparison, the winter bee pools had up-regulated medium term memory and visual behaviour as well as several genes in the pathway and hormone category. Of the latter, several are involved in regulation of food intake and nutrient sensing pathways, as for instance *neuropeptide F* (*npf*, GB16364, fold change 3.23) and *polarization-related protein LKB1* (*lkb1*, GB10693, fold change 5.48). Others are involved in the regulation of dopamine production, like *similar to Aromatic-L-amino-acid decarboxylase (AADC) (DOPA decarboxylase) (DDC)* (GB14019, fold change 5.84). The winter bee pools also showed a high investment in protein metabolism, processing, stability, maintenance and repair, with 34 genes in this category, many of which are involved in proteolysis. Two chaperone proteins were up-regulated, *similar to Protein lethal (2) essential for life (Protein Efl21)* (LOC724449, fold change 4.72) and *similar to lethal (3) 87Df CG7620-PA* (GB12668, fold change 4.49). Notably, the similar to *I'm not dead yet CG3979-PA, isoform A* (*indy*, GB15354, fold change 5.31), which has been shown to be a determinator of life span in many other species and which is a regulator of the body's energy storage and usage, is up-regulated in the winter bee pools. In the annotation DNA binding, metabolism, replication and repair, *homeotic protein caudal* (*cad*, GB10821, fold change 19.31), a regulator of development and cell division, the DNA repair enzymes *flap endonuclease 1* (*flap1*, GB15388, fold change 4.82) and *similar to tosca CG10387-PA* (LOC550962, fold change 4.40) were up-regulated. Notably, the genes encoding *similar to Histone H2B* (LOC726487, fold change 17.17) and the histone acyltransferase, *similar to CREB binding protein* (GB10171, fold change 3.95) are up-regulated, suggesting epigenetic regulation of gene expression. In the category immune defence, xenobiotic metabolism, stress response 11 expressed genes are up-regulated, many of which are involved in detoxification and defence against fungal infections. We found 13 transcription factors and 48 genes were annotated as unknown functions.

#### Foragers versus winter bee pools (figure 3c)

In the comparison of foragers and the winter bee pools, foragers showed an up-regulation of 17 genes encoding proteins involved in behaviour, sensory, learning and memory. The genes were typically involved in flight, in response to light, sound, mechanical and chemical stimuli, response to stress and ability for learning and memory. Cell growth and development contained 16 genes, and chromosome maintenance contained only similar *to corto CG2530-PA* (*corto*, GB13761, fold change 8.28), a general repressor of gene expression, suggesting a broad down-tuning of the protein synthesis machinery in foragers. Among the five expressed genes annotated as determination of adult life span was *similar to Probable G-protein coupled receptor Mth-like 1 precursor (Protein methuselah-like 1)* (GB11809, fold change 3.67), a known regulator of life span in Drosophila. Immune defence, xenobiotic metabolism, stress response contained 25 genes, several of these are antimicrobial peptides and others are main regulators of the Toll and IMD pathways. In the unknown function category, we also found *hypothetical LOC408807* (*irp30*, GB10708, fold change 15.12), which recently has been described as a typical secreted immune protein. Motor proteins and muscle contained 19 genes and pathways and hormones 18, most of which are involved in the process of producing dopamine and activation of MAPK activity. The 17 genes in protein metabolism, processing, stability, maintenance and repair were mostly genes involved in proteolysis. Transcription factors are a large group of 36 genes as were transport with 25, and the unknown function category with 47 genes.

#### Winter bee pools versus foragers (figure 3c)

In the reverse comparison, the winter bee pools had up-regulated two chaperones, *similar to Protein lethal l (2) essential for life (Protein Efl21)* (GB10339, fold change 4.42) and *similar to Protein lethal (2) essential for life (Protein Efl21)* (L OC724449, fold change 4.12) and had six genes in the term DNA binding, metabolism, replication and repair. In the latter group, the three DNA repair enzymes *similar to tosca CG10387-PA (tosca*, LOC550962, fold change 7.41), *flap endonuclease 1 (flap1*, GB15388, fold change 4.21) and *similar to O-6-alkylguanine-DNA alkyltransferase CG1303-PA* (LOC725876, fold change 3.58) were up-regulated. Two histones were also up-regulated in the winter bee pools. In the immune defence category, most genes are involved in stress response. Two mitochondrial ribosomal proteins and four nuclear ribosomal proteins were up-regulated, suggesting an increase increased synthesis of mitochondrial and nuclear proteins in this behavioural group. The group pathways and hormones contained *similar to CG12068-PA* (GB10499, fold change 6.06), which is involved in ecdysone biosynthetic process, and *eclosion hormone (eh*, GB19466, fold change 4.89). The odorant binding proteins *odorant binding protein 13 (obp13*, GB18363, fold change 4.05) and *odorant binding protein 14 (obp14*, NP_001035313.1, fold change 19.77) were also up-regulated. Protein metabolism, processing, stability, maintenance and repair had 16 expressed genes, many of which are proteases. Transcription held six expressed genes, and transferase activity four, including the *histone lysine methyltransferase similar to SET domain and mariner transposase fusion* (LOC726675, fold change 30.38) suggesting epigenetic regulation. Transport held nine expressed genes and the unknown function category 30.

#### Nurses versus vgknockdowns (figure 3d)

In comparison to *vg*knockdowns, nurses had a high expression level of *similar to 5-hydroxytryptamine 1A receptor (5-HT-1A) (Serotonin receptor 1A) (5-HT1A)* (GB17507, fold change 9.3) which is probably a modulator of social behaviour. Other genes with high expression levels encoding proteins possibly involved in social behaviour were *odorant binding protein 14* (*obp14*, NP_00103513.1, fold change 72.61), *odorant binding protein 1*3 (*obp13*, GB17875, fold change 10.57) and *chemosensory protein 1* (*csp1*, GB17875, fold change 12.28). The nurses also showed a high investment in the GO term cuticular proteins, chitinases and chitin metabolic process, where the main part of the proteins are involved in synthesis of cuticula or are structural proteins vital for the maintenance of the cuticula. The two proteins with the highest expression levels were the *cuticular protein 19* (*cp19*, GB 16692, fold change 118.15) and a protein which was designated as unknown functions and which may have a function in upholding cuticular structure, *hypothetical LOC552685* (GB11550, fold change 310.33). The nurses had an increased expression of the haemolymph and storage proteins *hexamerin 110* (*hex110*, GB14316, fold change 117.74) and *larval-specific very high density lipoprotein* (*vhdl*, GB15055, fold change 23.02).

#### Vgknockdowns versus nurses (figure 3d)

In the reverse comparison, *vg*knockdowns had five genes in the category autophagy and cell death, all with a fold change between 2.5 – 4.17. Most of the genes in the behaviour, sensory, learning and memory category were involved in adult feeding behaviour and sensory perception of chemical stimuli and sweet taste. The highest fold change was in *tyrosine hydroxylase* (*tyhyd*, GB15303, fold change 25.97) an enzyme responsible for production of L-DOPA, a precursor to dopamine. Other genes involved in the same process also had a high fold change *similar to homogentisate 1, 2-dioxygenase CG4779-PA* (LOC725911, fold change 31.72), *similar to Aromatic-L-amino-acid decarboxylase (AADC) (DOPA decarboxylase) (DDC)* (GB14019, fold change 22.11) which is the rate limiting step in the production of dopamine, as well as *phenylalanine hydroxylase* (GB18494, fold change 5.03) and *dopamine transporter* (*dat*, GB1526, fold change 4.75). Other genes in the category pathways and hormones were involved in TOR signalling, MAPK activation and regulation of renal activity. Most interesting were the up-regulation of the vitellogenin receptor, *yolkless* (*yl*, GB 16571, fold change 3.22) and the upstream kinase of AMPK, *polarization-related protein LKB1* (*lkb1*, GB10693, fold change 3.67.) Notably, there were six genes expressed in the category chaperone, with fold changes of 2.76 – 4.35. Among these were *heat shock protein Hsp70Ab-like* (*hsp70ab*, GB19503, fold change 2.76). There seemed to be a very high investment in immune defence, as this category had 31 members with a fold change of 2.58 – 97.64. The genes with the highest fold change were *defensin 1* (*def1*, GB19392, fold change 97.64) and *hypothetical LOC408807* (*irp30*, GB10708, fold change 77.21) in the unknown function category, which has newly been discovered to behave as a typical secreted immune protein in honey bees.

#### Nurses versus methoprene treated bees (figure 3e)

In comparison to methoprene treated bees, nurses showed a high investment in maintaining the cuticular structure with 15 expressed genes in this category. Most of the genes were involved in chitin metabolic process or are vital for maintenance of cuticular structure. The nurses also invest in protein synthesis as well as storage proteins.

#### Methoprene treated bees versus nurses (figure 3e)

In the opposite comparison, the methoprene treated bees had five expressed genes in the category autophagy and cell death and seven in the behaviour, sensory, learning and memory category. Most of the latter are involved in sensory perception. As in the *vg*knockdown comparison, this behavioural group also had up-regulated genes involved in the production of dopamine, positive regulation of the TOR signalling cascade, the vitellogenin receptor *yolkless*, MAPK activation and *lkb1*. Four histones and 14 chaperones were up-regulated in methoprene treated bees compared to nurses, as well as 26 in the immune defence category, most notably *defensin 1* (*def1*, GB19392, fold change 32.13) and *LOC408807* (*irp30*, GB10708, fold change 42.67), and 28 in protein metabolism, processing, stability, maintenance and repair. Most genes in the latter group are involved in proteolysis.

#### Nurses versus reversion bees (figure 3f)

In comparison to reversion bees, nurses showed a high investment in cuticular proteins, chitinase and chitin metabolic process with 36 expressed genes in this category with a fold change of 2.3–145.73. Most notable were *cuticular protein 19* (*cp19*, GB16692, fold change 145.73) and the possible cuticular protein *LOC552685* (GB11550, fold change 250.1) from the unknown function category. Nurses also showed a high investment in lipid, phospholipid, fatty acid metabolism and fatty acid beta oxidation with 20 expressed genes, most of them are involved in biosynthesis of phospholipids. Notably, both the haemolymph and storage proteins *vhdl* (GB15055, fold change 43.72), *hex110* (GB14361, fold change 78.27) and *hexamerin 70c* (*hex70c*, GB13613, fold change 6.3) were all up-regulated in nurses. The category transport also stood out with 35 expressed genes.

#### Reversion bees versus nurses (figure 3f)

In this comparison, reversion bees had four genes in the category autophagy and cell death. The genes involved in behaviour, sensory, learning and memory were mostly involved in sensory perception. As the *vg*knockdowns and the methoprene bees, the reversion bees also showed an up-regulation of genes involved in dopamine production, positive regulation of the TOR signalling cascade, *yolkless*, MAPK activation and *lkb1*, as well as chaperones and histones. The category immune defence had a high number of expressed genes with the two topmost were the same as in methoprene treated bees. Protein metabolism, processing, stability, maintenance and repair had 30 members, mostly proteases and genes involved in proteolysis. The reversion bees also invest in transcription, transferase activity and transport. All categories had a high number of expressed genes.

#### Foragers versus the manipulated worker groups (figure 3g, 3h and 3i)

In comparison to the manipulated groups, foragers showed a higher expression level in a large number of genes in the categories cell growth and development, transport and protein metabolism, processing, stability, maintenance and repair. The expressed genes in the latter category were mainly involved in proteolysis. The expressed gene with the overall highest fold change was *glycerol-3-phosphate dehydrogenase* (*gpdh*, GB11613, fold change in comparison to *vg*knockdowns 126.82, in comparison to methoprene treated bees 121.27, in comparison to reversion bees 42.86) an enzyme which in Drosophila is reported to be involved in TCA cycle and flight behaviour.

#### The manipulated worker groups versus foragers (figure 3g, 3h and 3i)

In comparison to foragers, *vg*knockdowns had a low total number of genes with a high fold change. *Vitellogenin* (*vg*, GB13999) had a fold change of 3.16, and the category pathways and hormones showed an up-regulation of genes involved in the production of dopamine and the heamolymph and storage protein *hex70b* (GB10869, fold change 3.17). In comparison to foragers, methoprene treated bees had 11expressed genes in the category chaperones and a 4.49 fold change in the expression level of *vg* (GB13999). The reversion bees showed an increase in investment in cuticular proteins, immune defence and protein metabolism in the comparison to foragers.

### Verification of transcriptome data by qPCR

The results from the qPCR were in accordance with the whole transcriptome analysis of RNA from the different honeybee worker phenotypes (table 1). There was a very good correlation between the experimental data and the qPCR in all comparisons apart from nurse bees versus *vg*knockdowns (table 1, correlation coefficients in the range of 0.79 to 0.93). The gene *vitellogenin* was utterly down-regulated in the *vg*knockdown samples and quite highly up-regulated in the nurse bee samples in the qPCR. Omitting this data point gave a correlation coefficient of 0.93435774 for the rest of the expressed genes in this comparison.

### Hierarchical clustering of functionally annotated subsets of genes

**Figure 4:**
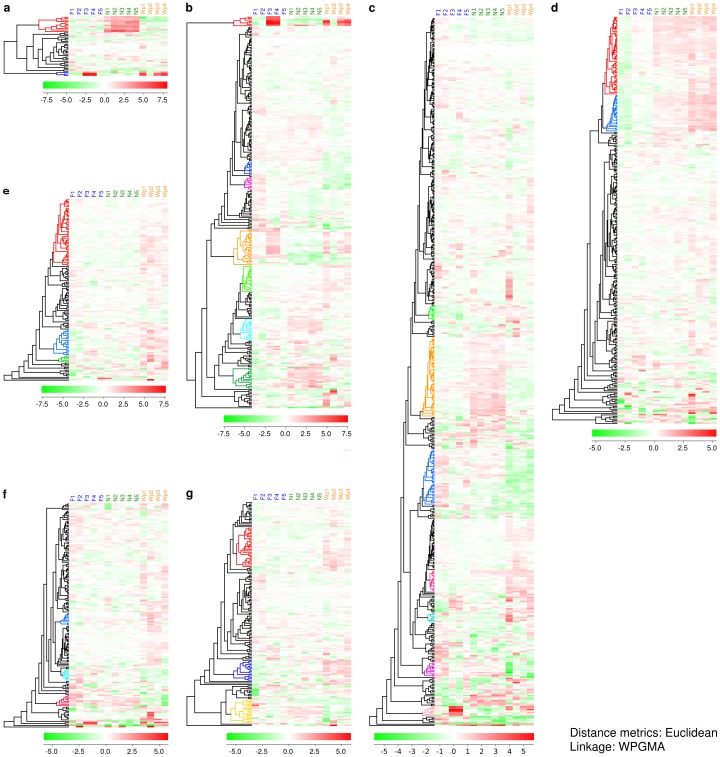
Heat maps of subset of functional categories with special relevance to ageing. a) cuticular proteins, chitinases and chitin metabolic process, b) immune defence, xenobiotic metabolism and stress response, c) pathways, behaviour, sensory, learning and memory, d) translation, e) chromosome maintenance and DNA repair, f) translation and g) cell growth and development. Clusters of specific interest are marked in colours. Abbreviations: (F1-5) foragers, (N1-5) nurses bee, (Wp1-5) winter bee pools.

The clustering analysis was performed for seven functional categories containing expressed genes of special relevance to ageing (figure 4, see also Table S3). The genes were chosen based on GO annotation from J-Express, NCBI, Flybase homologs and extensive literature mining. All categories showed interesting clusters with a clear difference in expression levels between nurses, foragers and winter bees.

In the category *cuticular proteins, chitinases and chitin metabolic process* the analysis revealed a small distinct cluster of 10 expressed genes that was up-regulated in nurses and not in foragers or winter bees (figure 4, a). Four of the expressed genes were cuticular protein analogous to peritrophins which are vital for cuticular structure; the rest was cuticular proteins and chitin deacetylases.

The category immune *defence, xenobiotic metabolism and stress response* showed seven clusters (figure 4, b). The first cluster (six genes) was up-regulated in two of the foragers and three of the winter bee pools. The main members in this cluster are involved in stress response and defence against fungal infections. The second (nine genes) and third (nine genes) clusters were down-regulated in the winter bee pools; the clusters contained genes that encode receptors in the Toll pathway as well as scavenger receptors. The fourth cluster contained 25 genes that were down-regulated in nurses. The cluster contained some antimicrobial peptides, cytochrome P450's and peptidoglycan recognition proteins. The fifth cluster (19 genes) was up-regulated in the winter bee pools. Most of the expressed genes in this cluster are involved in stress response. The sixth (15 genes) and seventh cluster (15 genes) were both up-regulated in nurses. The two clusters contained expressed genes involved in stress response, xenobiotic metabolism and also some antimicrobial peptides.

The category *pathways, hormones and behaviour* contained seven clusters (figure 4, c). The first cluster contained 10 genes that were down-regulated in foragers. The two most interesting genes in this cluster was *Raf kinase, effector of Ras* (*raf1*, GB16508) and *Sirt7histone deacetylase* (GB14227). The second cluster of 56 genes was up-regulated in nurses and contained several hormone receptors and genes encoding both metabolism and protection against oxidative stress. The third cluster was down-regulated in the winter bee pools and contained 39 genes. Several of the genes in this cluster are involved in the insulin-insulin like signalling pathway and others are involved in hormone synthesis pathways. The fourth cluster (15 genes) and the fifth (seven genes) were up-regulated in the winter bee pools; several of the expressed genes were involved in the insulin-insulin like signalling pathway or behaviour modulation. The two last clusters of ten and seven genes respectively contained genes involved in behaviour modulation. The first cluster was down-regulated in the winter bee pools and the last was down-regulated in nurses.

The *translation* term (figure 4, d) contained two large clusters down-regulated in foragers (55 and 25 genes respectively). Both clusters consisted of genes encoding ribosomal proteins and mitochondrial ribosomal proteins.

In the category *chromosome maintenance and DNA repair* (figure 4 e); there were three clusters (47, 18 and five genes respectively) that were all up-regulated in the winter bee pools.

The term *translation* had three clusters of nine; eight and eight genes respectively (figure 4, f). The first cluster was up-regulated in the winter bee pools whereas the two latter were down-regulated.

In the category *cell growth and development*, we found three clusters (figure 4, g). The two first clusters were up-regulated in the winter bee pools whereas the latter were down-regulated.

### Fold change of genes in the insulin-insulin like signalling pathway and accessory pathways

Expressed genes were selected on the basis of Kegg (http://www.genome.jp/kegg/) insulin like signalling pathway map, GO annotation from J-Express, NCBI, Flybase homologs and extensive literature mining. The chosen genes were compared with respect to fold change between nurse bees and foragers, and nurse bees and winter bees. Only genes with a fold change higher than 2 were considered (Table 3).

**Table 3:** Expressed genes within the insulin-insulin like signalling pathway.

Sample	Gene name	GB number	Fold change
**F vs. N**	Pepck	GB16196	5,54
	Pka-C3	GB14368	3,84
	LOC411959	GB16883	3,44
	lkb1	GB10693	2,24
	Vkr	GB15898	2,2
	Protein kinase 61C	GB15780	2,1
	Dsor1	GB13922	2,0
**N vs. F**	GlyP	GB11892	2,4
	Raf1	GB16508	2,3
	ILP-2	GB10174	2,1
	S6	GB18619	2,1
**Wb vs. N**	lkb1	GB10693	8,8
	similar to CG9238	GB12349	3,8
	rl	GB15499	2,3
	exo70	LOC551576	2,1
	GβL	GB19722	2,1
	Tor	GB11213	2,1
	Cbl	GB16551	2,0
**N vs. Wb**	Glycogen binding subunit 76A	GB14703	7,6
	IGF-I receptor	LOC725827	4,7
	SNF1A	GB15108	4,3
	Hexokinase	GB19387	3,1
	bsk	GB16401	3,1
	HIF-1 alpha	GB16786	2,8
	Protein kinase 61C CG1210-PC, isoform C	GB15780	2,7
	Pka-C3	GB14368	2,5
	drk	GB15989	2,2
	IR-2	GB18331	2,1
	Pka-C1	GB17175	2,1

Comparison between worker phenotypes, only expressed genes with a fold change above 2 was considered. Abbreviations: F (foragers), N (nurse bees), Wb (winter bees).

## Discussion

In this work we have analysed a large data set at three different levels. Both the unbiased tests and the focused clustering analysis of the subsets of data with special relevance to somatic maintenance, pointed in the same direction (figure 2 and 4; Tables S1–S3). From the GO analysis we saw a shift in investment between nurses and foragers, where nurses seemed to invest in somatic maintenance and energy production and foragers in immune defence and protein metabolism (figure 2 and 4, Tables S1–S3). In comparison to winter bees, this shift was even more pronounced. The comparison of foragers and winter bees showed a high investment in energy production in foragers and a more even-paced investment in all categories in winter bees (figure 2 and 4, Tables S1–S3). Both *vg*knockdowns and methoprene treated bees showed a similar pattern in expressed genes as the foragers, although the results were not consistent across the sampled individuals. The reversion bees also tended to show the same pattern in expression as foragers, possibly reflecting that they were not fully reverted to being nurses which may be due to the early sampling after reversion. To delve deeper into our data set, we looked closely at selected categories of genes with special relevance to somatic maintenance and ageing. The expressed genes in each category were chosen from extensive literature mining and homology searches and were analysed by hierarchical clustering (figure 4, table S3).

### Cuticular proteins, chitin and chitin metabolic process

For all insects cuticular proteins and chitinases are of vital importance, not only in restricting water loss; but also in digestion, arthropod moulting, defence/immunity and resistance to pathogens [18]. Nurses consistently showed a higher number of genes highly expressed in this functional category than all other worker phenotypes (figure 4, Tables S2–S3). Specifically, cuticular proteins analogous to peritrophins, cuticular proteins and chitin deacetylases were high in nurses compared to foragers and the winter bee pools (figure 4, Table S3). The nurses need to maintain chitin bearing structures may be due to dietary differences. The primary means by which the nutrients from pollen are made available to the colony is by conversion to jelly by nurse bees [19]. The constant need to convert protein and fat into jelly would put the digestive system under pressure and maintenance of a solid structure would be of more importance to nurses than to foragers and winter bees. Nurses are also subjected to wear and tear: recognition and cleaning, “licking”, to keep the hive free of parasites and microorganisms. This load is lower during winter when there is no transport of external material into the hive and no larvae; and evaporation of water is reduced since the temperature is lower. Thus, the investment in somatic maintenance at one crucial level, the body armour, is higher in nurses than in the two other phenotypes.

### Immune defence, xenobiotic metabolism and stress response

In the GO analysis both nurses and foragers, as well as the manipulated groups, showed an up-regulation of expression in this term (figure 4, tables S1–S3). The clustering analysis revealed that both foragers and nurses invest in xenobiotic metabolism and stress response, whereas winter bees seem to up-regulate antimicrobial humoral responses (figure 4, table S1–S3). In the comparisons with a cut-off of q = 10%, foragers had a very high expression level of the novel [20] immune responsive protein IRP30 (*Hypothetical LOC408807*) in comparison to both nurses and the winter bee pools. The same held true in the comparisons of nurses and the two manipulated groups, *vg*knockdown and methoprene treated bees. This protein has been thought to be specific for social insects [20] and our results indicate that it may play an important role in the forager phenotype. Xenobiotic metabolism is important in the detoxification of environmental pollutants, such as different pesticides and herbicides that a bee may encounter on foraging trips, or by the consummation of nectar and pollen [21]. Thus, a high investment in detoxification of external pollutants is necessary. Flying honeybees have among the highest mass specific metabolic rates ever measured, suggesting that their flight muscles may experience high levels of oxidative stress during normal daily activities. Mitochondrial DNA damage leads to disruption of the electron transport chain and production of more radical oxygen species. One would assume that free radical defence would be a priority in this worker phenotype. However, as our data only shows the expressed genes in the worker fat body, the picture may have been different if muscle tissue had been included. During winter, bees stay in a cluster while maintaining a resting metabolism [22]. The shift to a predominantly antimicrobial humoral response is presumably due to the stability of the environment and the lack of external influence. This shift is another example of making only the most necessary investment to withstand the actual environmental pressure.

An important mechanism in stress protection of proteins and improving their ability to function properly, are the chaperones [23], [24]. The expression of genes encoding chaperones showed some very distinct differences especially between the manipulated and the natural groups. Between the natural groups, there were only one or two genes that were significantly differentially expressed. Reversion bees (foragers on their way to become nurses again) had four chaperones up-regulated compared to nurses, *vg*knockdowns (nurses forced to become foragers) had six and methoprene treated bees (nurses forced to become foragers) had 14 genes up compared to nurses. Compared to foragers, 11 genes were up-regulated in methoprene-treated bees. This up-regulation in either direction suggests that high levels of juvenile hormone, mimicked by methoprene, increases synthesis of chaperone proteins to protect proteins and increase accuracy and robustness in transitions between life stages and probably also during development.

### Nutrition and growth

Compared to both foragers and winter bees, nurses had a higher number of expressed genes in lipid metabolism and a lower number in carbohydrate metabolism (figure 4; tables S2–S3). This presumably reflects that the diet of both foragers and winter bees are predominantly carbohydrates, whereas the nurses are the pollen-to-jelly converters of the hive. Although the comparison between nurses and foragers showed a higher number of expressed genes in the category protein metabolism in foragers, a closer look revealed a vital difference in the proteins expressed. Foragers expressed phosphatases, proteases and peptidases; and nurses expressed genes involved with processing, modification and maturation. In the comparison with a cut off of q = 10%, nurses showed a higher investment in genes encoding the haemolymph and storage proteins *vitellogenin* (GB13999), *larval-specific very high density lipoprotein* (GB15055) and *hexamerin 110* (GB14361) than both foragers, *vg*knockdowns, methoprene treated bees and reversion bees. Whereas foragers, *vg*knockdowns, methoprene treated bees and the reversion bees all showed a high proteolysis activity. This result probably reflects a degradation of protein storage in the forager fat body. As a preparation to foraging, workers use their fat bodies as a source of energy, fat and proteins and thereby reduce their demand of nutrients from the hive. Protein synthesis is one of the most energy consuming cellular processes, devouring an estimated 50% of total cellular energy [25]. Limiting the protein synthesis in foragers therefore seems like a good energy saving solution on both the individual and colony level. A reduced translation rate would reduce not only normal protein production but also the flux of damaged proteins through various pathways [26].

### Cell growth, chromosome maintenance and DNA repair

These functional terms were up-regulated in winter bees only (figure 4, table S3). All main DNA repair pathways were up-regulated. The massive up-regulation probably reflects an increase in DNA repair per se, in addition to an escalation in DNA synthesis as a result of cell growth. It is also notable that genes encoding telomerase binding proteins and telomerase reverse transcriptases showed a high up-regulation in winter bees. The significance of DNA repair and chromosome maintenance, including telomeres, in ageing and regulation of life span is well documented [27], [28], [29], [30], [31]. These results show that genomic and cellular maintenance are given priority in the winter bees, whose survival over the winter months is of vital importance to the continued life of the honeybee colony the following spring. This is directly opposed to the investments in the individual workers in the summer season, when individuals are exposed to high environmental risks and are rapidly replaced with new individuals when they die.

### Pathways, behaviour, sensory, learning and memory

In the unsupervised clustering analysis a large selection of genes were expressed in this category (figure 4, Tables S1–S3). The GO analysis with a cut-off of q = 10% and the supervised clustering also revealed a similar pattern to the unsupervised clustering analysis. Although these genes are not so directly linked to somatic maintenance as the other functional groups, they are crucial for survival and the priorities are illuminating. Nurses and foragers up-regulate genes in connection with long term memory and adult locomotory behaviour. Winter bees on the other hand, express genes involved in visual stimuli and courtship behaviour. Compared to nurses and winter bees, foragers down-regulate genes involved in sensory perception of sound, pain and response to starvation. Winter bees down-regulate visual stimuli, response to light and positive regulation of sleep/wake cycle. These genes may not be crucial for winter workers since they do not leave the hive during the winter months. The odorant binding proteins showed a marked difference in expression between the different behavioural groups. Apart from the comparison with the winter bee pools, nurses showed an up-regulation of *odorant binding protein 14* (NP.001035313.1) in all other comparisons. The winter bee pools also had synthesis of Obp 14 up-regulated in comparison to foragers, suggesting that this protein may have an importance in the social environment of the hive. We observed a consistent high expression of the *serotonin receptor 1 A* (*5ht1a*, GB17507) in nurses (figure 1 and 2, Tables S1–S2) in comparison to foragers and also in *vg*knockdowns. In *Caenorhabditis elegans,* serotonin signals a positive event like food or mate [32]. In *Drosophila*, *5ht1a* is involved in response to light [33] and aggressive behaviour [34] as well as insulin production [35]. Serotonin is involved in circadian rhythms, locomotion, feeding, learning and memory in invertebrates [36]. In humans, genetic variations and dysregulations in the *5-HT1A* homolog are linked to depression, anxiety and schizophrenia [37], [38]. It may be that this gene is vital to nurses as they live in a strict social context and must be able to respond to small social cues, whereas foragers spending most of the day on their own need to make appropriate behavioural responses relying mainly on own judgement. Both foragers, *vg*knockdowns and methoprene treated bees showed an up-regulation in genes involved in the production of dopamine, positive regulation of the TOR signalling pathway, the *vg* receptor *yolkless*, MAPK activation and *lkb1* in comparison to nurses. Dopamine plays a major role in the brain system responsible for reward-driven learning and it is perhaps not surprising that this is up-regulated in foragers and the manipulated groups that are precocious foragers. The up-regulation of *yolkless* may reflect a mobilization of stored vitellogenin from the fat body. TOR signalling integrates the input from upstream pathways including insulin-insulin like signalling, growth factors and amino acids as well as being a modulator of cellular nutrient, oxygen and energy levels. An increase in these pathways possibly reflects the higher need for energy involved in foraging activities (see also table 3). It is striking that so many genes crucial for behaviouristic phenotypes are expressed in the fat body. Hence, the fat body probably has a support function for the brain.

### Implications and final conclusions

Honeybees are eusosial insects where the fundamental components that underlie superorganismal order are the female forms [4]. In a social insect superorganism, the non-reproducing workers can be regarded as an extended form of the reproductive queen's soma. Like sterile somatic cells, the workers differentiate and communicate to produce coordinated patterns of growth, homeostasis, provisioning and defence before death [4], and the division of labour are like different tissues in a body with different tasks. In this study, we observed that genes encoding functions crucial for somatic maintenance of the individual bee were expressed in a pattern so that that preferentially the most threatening challenges, not only for the bee but just as much or perhaps more for the hive, are met. Crucial functions are spared whereas others are down-tuned to a functionally basic or minimum level for the remaining individual lifespan to be expected from the external challenges the bee will met at its actual life stage. Survival of a reproducing organism is the result of the capacity to withstand challenges from external and internal sources. If the bee society functions as a superorganism, the observed patterns are exactly what could be expected for the non-reproducing bees, considering them as a disposable soma of this organism.

To illuminate the certainly many molecular mechanisms of these different maintenance patterns has been beyond the scope of this paper. The expression patterns of genes involved in chromatin structure, histone functions and DNA methylation, do however suggest that epigenetic mechanisms are involved in the massive down-regulation of almost all somatic maintenance in foragers.

## Supporting Information

Figure S1 Eksample of qPCR plate set up for verification of whole transcriptome data.
(DOCX)

File S1 Supplementary tables S5–13: Median fold change values, minimum and maximum values within each GO annotation term of the comparison of the behavioural groups with a cut off of 10%.
(DOCX)

Table S1 Expressed genes in each of the clusters from the hierarchical clustering displayed in figure 2.
(XLSX)

Table S2 Expressed genes with a Rank test value of q = 10% in comparisons of all worker phenotypes.
(XLSX)

Table S3 Expressed genes in clusters from hierarchical clustering of functional categories with special relevance to aging. The resulting heat maps are displayed in figure 4.
(XLSX)

Table S4 Primer design for qPCR analysis.
(DOCX)
