# Supplementary material for: Somatic Maintenance Resources in the Honeybee Worker Fat Body Are Distributed to Withstand the Most Life-Threatening Challenges at Each Life Stage
Source: PLoS One. 2013 Aug 5;8(8):e69870. doi: 10.1371/journal.pone.0069870 (PMC3734224; doi:10.1371/journal.pone.0069870)
Supplement: Figure S1 — Eksample of qPCR plate set up for verification of whole transcriptome data. (DOCX) [file pone.0069870.s001.docx]

*Supplementary figure 1: Eksample of qPCR plate set up for verification of whole transcriptome data*
