# Supplementary material for: Somatic Maintenance Resources in the Honeybee Worker Fat Body Are Distributed to Withstand the Most Life-Threatening Challenges at Each Life Stage
Source: PLoS One. 2013 Aug 5;8(8):e69870. doi: 10.1371/journal.pone.0069870 (PMC3734224; doi:10.1371/journal.pone.0069870)
Supplement: File S1 — Supplementary tables S5–13: Median fold change values, minimum and maximum values within each GO annotation term of the comparison of the behavioural groups with a cut off of 10%. (DOCX) [file pone.0069870.s002.docx]

| Supplementary table 5 | Nurses vs foragers | | | Foragers vs nurses | | |
| --- | --- | --- | --- | --- | --- | --- |
| GO annotation | Median | Mina | Maxa | Median | Mina | Maxa |
| Autophagy, apoptosis and cell death | 5,8 | 5,8 | 5,8 | 6,9 | 3,0 | 10,9 |
| Behaviour, sensory, learning and memory | 3,3 | 3,0 | 4,9 | 2,9 | 1,5 | 5,6 |
| Binding | 3,7 | 2,7 | 12,9 | 3,1 | 2,5 | 3,8 |
| Carbohydrate metabolic process | 3,5 | 2,8 | 4,3 | 4,6 | 2,5 | 8,1 |
| Cell growth and development | 10,5 | 3,1 | 45,4 | 9,8 | 7,5 | 12,1 |
| Chaperone | 0 | 0 | 0 | 3,5 | 3,5 | 3,5 |
| Chromosome maitenance | 0 | 0 | 0 | 0 | 0 | 0 |
| Cuticular proteins, chitinase and chitin metabolic process | 5,0 | 1,6 | 90,6 | 8,5 | 4,7 | 30,2 |
| Determination of adult life span | 3,5 | 2,6 | 12,9 | 3,9 | 2,9 | 4,8 |
| DNA binding, metabolism, replication and repair | 5,4 | 2,6 | 9,7 | 2,8 | 2,8 | 2,9 |
| Energy | 4,7 | 3,5 | 7,2 | 4,4 | 2,8 | 19,3 |
| Histone | 0 | 0 | 0 | 3,6 | 3,6 | 3,6 |
| Immune defence, xenobiotic metabolism, stress response | 3,7 | 2,6 | 11,0 | 5,2 | 2,6 | 24,1 |
| Kinase | 3,8 | 3,0 | 10,5 | 5,5 | 4,3 | 6,7 |
| Lipid, phospholipid, fatty acid metabolism and fatty acid beta oxidation | 4,3 | 2,7 | 17,8 | 3,7 | 2,6 | 11,5 |
| Membrane | 3,7 | 3,5 | 3,8 | 6,7 | 3,1 | 12,9 |
| Mitochondrial proteins and mitochondrial ribosomal proteins | 4,9 | 2,8 | 5,6 | 3,1 | 3,1 | 3,1 |
| Motor proteins and muscle | 4,1 | 2,5 | 8,9 | 4,8 | 2,9 | 21,5 |
| Neurogenesis | 3,4 | 2,9 | 9,4 | 4,4 | 3,4 | 7,2 |
| Nuclar ribosomal proteins | 0 | 0 | 0 | 0 | 0 | 0 |
| Odorant binding proteins and receptors | 10,3 | 3,2 | 72,5 | 2,8 | 2,8 | 2,8 |
| Pathways and hormones | 4,5 | 2,6 | 25,0 | 4,3 | 2,7 | 10,5 |
| Protein metabolism, processing, stability, maitenance and repair | 3,5 | 2,6 | 4,1 | 7,1 | 2,9 | 22,9 |
| Receptor activity | 3,7 | 3,5 | 3,9 | 3,2 | 3,2 | 3,2 |
| Signaling | 4,1 | 2,7 | 5,5 | 5,0 | 4,4 | 5,6 |
| TCA | 0 | 0 | 0 | 0 | 0 | 0 |
| Transcription | 3,3 | 2,8 | 3,6 | 3,8 | 2,6 | 13,5 |
| Transferase activity | 3,2 | 2,6 | 14,8 | 4,8 | 2,7 | 27,0 |
| Translation | 0 | 0 | 0 | 0 | 0 | 0 |
| Transport | 4,2 | 2,6 | 7,6 | 4,7 | 2,7 | 16,1 |
| Unknown function | 3,8 | 2,6 | 191,9 | 4,0 | 2,6 | 63,3 |

**Supplementary tables 5 - 13**

| Supplementary table 6 | Nurses vs wp | | | Wp vs nurses | | |
| --- | --- | --- | --- | --- | --- | --- |
| GO annotation | Median | Mina | Maxa | Median | Mina | Maxa |
| Autophagy, apoptosis and cell death | 0 | 0 | 0 | 5,2 | 4,1 | 6,4 |
| Behaviour, sensory, learning and memory | 0 | 0 | 0 | 5,3 | 3,5 | 5,3 |
| Binding | 10,0 | 8,1 | 28,1 | 3,5 | 3,2 | 11,3 |
| Carbohydrate metabolic process | 0 | 0 | 0 | 19,9 | 5,6 | 34,8 |
| Cell growth and development | 12,3 | 8,5 | 16,1 | 4,5 | 3,7 | 9,3 |
| Chaperone | 0 | 0 | 0 | 4,6 | 4,5 | 4,7 |
| Chromosome maitenance | 0 | 0 | 0 | 4,0 | 3,5 | 5,5 |
| Cuticular proteins, chitinase and chitin metabolic process | 17,3 | 7,8 | 118,2 | 7,0 | 3,0 | 53,3 |
| Determination of adult life span | 0 | 0 | 0 | 5,3 | 5,3 | 5,3 |
| DNA binding, metabolism, replication and repair | 9,2 | 8,7 | 9,7 | 4,6 | 3,4 | 19,3 |
| Energy | 0 | 0 | 0 | 3,2 | 3,2 | 3,2 |
| Histone | 0 | 0 | 0 | 4,9 | 3,9 | 17,2 |
| Immune defence, xenobiotic metabolism, stress response | 0 | 0 | 0 | 5,7 | 3,1 | 45,8 |
| Kinase | 0 | 0 | 0 | 8,8 | 5,9 | 48,1 |
| Lipid, phospholipid, fatty acid metabolism and fatty acid beta oxidation | 9,8 | 8,9 | 10,6 | 8,3 | 3,9 | 27,5 |
| Membrane | 0 | 0 | 0 | 7,6 | 3,4 | 11,7 |
| Mitochondrial proteins and mitochondrial ribosomal proteins | 0 | 0 | 0 | 0 | 0 | 0 |
| Motor proteins and muscle | 11,0 | 8,2 | 13,8 | 4,0 | 3,0 | 5,8 |
| Neurogenesis | 14,4 | 14,4 | 14,4 | 5,0 | 3,2 | 7,7 |
| Nuclar ribosomal proteins | 0 | 0 | 0 | 4,9 | 4,5 | 5,3 |
| Odorant binding proteins and receptors | 0 | 0 | 0 | 3,4 | 3,3 | 3,5 |
| Pathways and hormones | 9,2 | 8,4 | 54,4 | 4,8 | 3,2 | 7,5 |
| Protein metabolism, processing, stability, maitenance and repair | 0 | 0 | 0 | 7,7 | 3,1 | 84,7 |
| Receptor activity | 0 | 0 | 0 | 3,8 | 3,5 | 4,0 |
| Signaling | 0 | 0 | 0 | 3,9 | 3,6 | 29,8 |
| TCA | 0 | 0 | 0 | 11,4 | 8,5 | 14,3 |
| Transcription | 9,6 | 9,6 | 9,6 | 4,1 | 3,1 | 13,8 |
| Transferase activity | 11,3 | 11,3 | 11,3 | 4,1 | 3,8 | 38,0 |
| Tranlation | 0 | 0 | 0 | 0 | 0 | 0 |
| Transport | 11,8 | 9,5 | 14,9 | 5,0 | 3,4 | 27,6 |
| Unknown function | 11,7 | 7,5 | 408,7 | 4,9 | 3,0 | 27,0 |

| Supplementary table 7 | Foragers vs wp | | | Wp vs foragers | | |
| --- | --- | --- | --- | --- | --- | --- |
| GO annotation | Median | Mina | Maxa | Median | Mina | Maxa |
| Autophagy, apoptosis and cell death | 4,3 | 3,2 | 6,5 | 0 | 0 | 0 |
| Behaviour, sensory, learning and memory | 4,6 | 3,3 | 45,8 | 5,3 | 5,3 | 5,4 |
| Binding | 4,1 | 2,8 | 5,4 | 4,7 | 4,4 | 7,2 |
| Carbohydrate metabolic process | 3,7 | 3,4 | 4,4 | 3,6 | 2,7 | 4,0 |
| Cell growth and development | 4,1 | 2,6 | 9,6 | 4,9 | 3,8 | 13,3 |
| Chaperone | 0 | 0 | 0 | 4,3 | 4,1 | 4,4 |
| Chromosome maitenance | 8,3 | 8,3 | 8,3 | 4,5 | 3,8 | 5,4 |
| Cuticular proteins, chitinase and chitin metabolic process | 5,6 | 3,4 | 7,4 | 4,7 | 2,6 | 6,4 |
| Determination of adult life span | 3,7 | 3,4 | 4,8 | 5,7 | 5,7 | 5,7 |
| DNA binding, metabolism, replication and repair | 4,1 | 3,4 | 13,4 | 4,2 | 3,6 | 10,0 |
| Energy | 18,5 | 18,5 | 18,5 | 0 | 0 | 0 |
| Histone | 0 | 0 | 0 | 5,1 | 5,4 | 5,4 |
| Immune defence, xenobiotic metabolism, stress response | 4,2 | 3,3 | 10,2 | 4,1 | 3,6 | 8,7 |
| Kinase | 0 | 0 | 0 | 6,7 | 6,2 | 7,2 |
| Lipid, phospholipid, fatty acid metabolism and fatty acid beta oxidation | 4,0 | 3,5 | 5,1 | 3,8 | 3,7 | 4,5 |
| Membrane | 3,7 | 3,3 | 4,7 | 6,7 | 4,7 | 8,8 |
| Mitochondrial proteins and mitochondrial ribosomal proteins | 0 | 0 | 0 | 5,4 | 3,5 | 7,2 |
| Motor proteins and muscle | 4,1 | 2,8 | 11,0 | 5,0 | 4,0 | 9,1 |
| Neurogenesis | 4,4 | 4,0 | 6,0 | 4,7 | 4,4 | 5,0 |
| Nuclear ribosomal protein | 0 | 0 | 0 | 4,7 | 4,1 | 5,9 |
| Odorant binding proteins and receptors | 0 | 0 | 0 | 11,9 | 4,1 | 19,8 |
| Pathways and hormones | 4,4 | 3,4 | 11,7 | 4,5 | 3,8 | 6,1 |
| Protein metabolism, processing, stability, maitenance and repair | 4,8 | 3,6 | 8,7 | 4,1 | 2,3 | 7,5 |
| Receptor activity | 5,8 | 3,9 | 9,2 | 0 | 0 | 0 |
| Signaling | 3,9 | 3,5 | 5,9 | 4,8 | 4,8 | 4,8 |
| TCA | 4,1 | 4,1 | 4,1 | 3,1 | 3,1 | 3,1 |
| Transcription | 4,4 | 2,8 | 7,0 | 3,8 | 3,3 | 5,9 |
| Transferase activity | 4,4 | 3,0 | 7,2 | 8,2 | 6,8 | 30,4 |
| Translation | 4,5 | 4,1 | 4,9 | 0 | 0 | 0 |
| Transport | 4,6 | 3,3 | 12,7 | 4,6 | 3,5 | 8,3 |
| Unknown function | 4,6 | 2,6 | 22,5 | 4,3 | 2,3 | 8,4 |

| Supplementary table 8 | Nurses vs v*g*knockdowns | | | *vg*knockdowns vs nurses | | |
| --- | --- | --- | --- | --- | --- | --- |
| GO annotation | Median | Mina | Maxa | Median | Mina | Maxa |
| Autophagy, apoptosis and cell death | 0 | 0 | 0 | 3,4 | 2,5 | 4,2 |
| Behaviour, sensory, learning and memory | 9,3 | 9,3 | 9,3 | 3,5 | 2,7 | 26,0 |
| Binding | 9,7 | 7,8 | 10,4 | 3,2 | 2,7 | 4,3 |
| Carbohydrate metabolic process | 25,7 | 25,7 | 25,7 | 4,1 | 3,6 | 10,7 |
| Cell growth and development | 15,1 | 11,1 | 23,0 | 3,2 | 2,8 | 31,7 |
| Chaperone | 0 | 0 | 0 | 3,0 | 2,8 | 4,4 |
| Chromosome maitenance | 0 | 0 | 0 | 0 | 0 | 0 |
| Cuticular proteins, chitinase and chitin metabolic process | 14,7 | 7,1 | 118,2 | 4,9 | 2,7 | 37,9 |
| Determination of adult life span | 0 | 0 | 0 | 2,6 | 2,6 | 2,6 |
| DNA binding, metabolism, replication and repair | 9,9 | 9,9 | 9,9 | 3,0 | 2,3 | 6,2 |
| Energy | 0 | 0 | 0 | 19,6 | 2,6 | 36,7 |
| Histone | 0 | 0 | 0 | 4,3 | 3,2 | 5,4 |
| Immune defence, xenobiotic metabolism, stress response | 11,2 | 10,4 | 13,5 | 4,7 | 2,6 | 97,6 |
| Kinase | 0 | 0 | 0 | 3,1 | 2,6 | 3,9 |
| Lipid, phospholipid, fatty acid metabolism and fatty acid beta oxidation | 23,0 | 9,4 | 27,6 | 3,3 | 2,6 | 4,5 |
| Membrane | 25,2 | 25,2 | 25,2 | 3,5 | 2,1 | 5,7 |
| Mitochondrial proteins and mitochondrial ribosomal proteins | 0 | 0 | 0 | 0 | 0 | 0 |
| Motor proteins and muscle | 6,5 | 6,5 | 6,5 | 3,5 | 2,7 | 32,5 |
| Neurogenesis | 54,8 | 54,8 | 54,8 | 3,1 | 2,9 | 4,8 |
| Nuclear ribosomal protein | 0 | 0 | 0 | 0 | 0 | 0 |
| Odorant binding proteins and receptors | 72,6 | 72,6 | 72,6 | 3,1 | 3,1 | 3,1 |
| Pathways and hormones | 12,3 | 7,9 | 117,7 | 3,7 | 2,7 | 22,1 |
| Protein metabolism, processing, stability, maitenance and repair | 0 | 0 | 0 | 3,9 | 2,4 | 33,8 |
| Receptor activity | 7,2 | 7,2 | 7,2 | 2,9 | 2,5 | 3,2 |
| Signaling | 0 | 0 | 0 | 3,2 | 2,6 | 3,2 |
| TCA | 0 | 0 | 0 | 4,0 | 3,2 | 4,7 |
| Transcription | 7,7 | 7,7 | 7,7 | 3,5 | 2,5 | 21,1 |
| Transferase activity | 9,5 | 9,5 | 9,5 | 4,4 | 2,5 | 28,8 |
| Translation | 0 | 0 | 0 | 4,2 | 3,7 | 8,5 |
| Transport | 12,6 | 6,8 | 20,5 | 3,3 | 2,5 | 16,9 |
| Unknown function | 25,0 | 6,5 | 310,3 | 3,3 | 2,4 | 77,2 |

| Supplementary table 9 | Nurses vs methoprene | | | Methoprene vs nurses | | |
| --- | --- | --- | --- | --- | --- | --- |
| GO annotation | Median | Mina | Maxa | Median | Mina | Maxa |
| Autophagy, apoptosis and cell death | 0 | 0 | 0 | 3,3 | 2,6 | 4,6 |
| Behaviour, sensory, learning and memory | 0 | 0 | 0 | 3,7 | 2,8 | 16,5 |
| Binding | 38,1 | 18,4 | 57,8 | 3,5 | 2,9 | 4,1 |
| Carbohydrate metabolic process | 11,8 | 11,8 | 11,8 | 3,6 | 2,5 | 4,7 |
| Cell growth and development | 7,6 | 6,1 | 18,6 | 3,9 | 2,7 | 14,8 |
| Chaperone | 0 | 0 | 0 | 6,4 | 2,7 | 20,4 |
| Chromosome maitenance | 0 | 0 | 0 | 2,8 | 2,8 | 2,8 |
| Cuticular proteins, chitinase and chitin metabolic process | 10,6 | 5,9 | 127,9 | 5,8 | 2,7 | 11,0 |
| Determination of adult life span | 0 | 0 | 0 | 2,6 | 2,5 | 2,7 |
| DNA binding, metabolism, replication and repair | 0 | 0 | 0 | 2,7 | 2,4 | 6,3 |
| Energy | 0 | 0 | 0 | 14,2 | 3,9 | 24,6 |
| Histone | 0 | 0 | 0 | 4,0 | 1,9 | 5,2 |
| Immune defence, xenobiotic metabolism, stress response | 7,4 | 5,7 | 18,1 | 4,1 | 2,5 | 32,1 |
| Kinase | 0 | 0 | 0 | 3,5 | 2,7 | 4,3 |
| Lipid, phospholipid, fatty acid metabolism and fatty acid beta oxidation | 8,6 | 5,3 | 22,2 | 2,9 | 2,6 | 5,9 |
| Membrane | 9,8 | 9,8 | 9,8 | 3,8 | 3,3 | 4,7 |
| Mitochondrial proteins and mitochondrial ribosomal proteins | 9,2 | 9,2 | 9,2 | 2,8 | 2,7 | 3,7 |
| Motor proteins and muscle | 0,0 | 0,0 | 0,0 | 2,9 | 1,6 | 22,8 |
| Neurogenesis | 12,6 | 12,6 | 12,6 | 3,4 | 2,5 | 8,3 |
| Nuclear ribosomal protein | 0 | 0 | 0 | 0 | 0 | 0 |
| Odorant binding proteins and receptors | 9,3 | 6,9 | 18,4 | 0 | 0 | 0 |
| Pathways and hormones | 7,7 | 5,6 | 16,3 | 3,4 | 2,4 | 11,0 |
| Protein metabolism, processing, stability, maitenance and repair | 6,0 | 6,0 | 6,0 | 3,7 | 2,2 | 11,7 |
| Receptor activity | 7,2 | 7,2 | 7,2 | 3,0 | 3,0 | 3,0 |
| Signaling | 0 | 0 | 0 | 2,7 | 2,5 | 3,6 |
| TCA | 0 | 0 | 0 | 53,2 | 53,2 | 53,2 |
| Transcription | 0 | 0 | 0 | 2,8 | 2,4 | 37,8 |
| Transferase activity | 7,7 | 5,4 | 10,0 | 3,2 | 2,1 | 20,2 |
| Translation | 0 | 0 | 0 | 5,3 | 5,3 | 5,3 |
| Transport | 8,8 | 7,7 | 14,3 | 3,7 | 2,6 | 17,4 |
| Unknown function | 10,5 | 5,7 | 98,5 | 3,2 | 2,4 | 42,7 |

| Supplementary table 10 | Nurses vs reversion | | | Reversion vs nurses | | |
| --- | --- | --- | --- | --- | --- | --- |
| GO annotation | Median | Mina | Maxa | Median | Mina | Maxa |
| Autophagy, apoptosis and cell death | 3,9 | 3,8 | 3,9 | 3,6 | 2,3 | 9,6 |
| Behaviour, sensory, learning and memory | 5,9 | 2,4 | 7,6 | 2,8 | 2,3 | 29,3 |
| Binding | 4,0 | 2,3 | 27,8 | 2,6 | 2,3 | 2,7 |
| Carbohydrate metabolic process | 4,2 | 2,9 | 13,5 | 3,5 | 2,5 | 5,2 |
| Cell growth and development | 3,1 | 2,2 | 66,3 | 3,3 | 2,2 | 46,8 |
| Chaperone | 2,4 | 2,4 | 2,4 | 3,1 | 2,2 | 4,3 |
| Chromosome maitenance | 0 | 0 | 0 | 0 | 0 | 0 |
| Cuticular proteins, chitinase and chitin metabolic process | 4,2 | 2,3 | 145,7 | 6,8 | 2,3 | 22,4 |
| Determination of adult life span | 2,9 | 2,3 | 7,7 | 0 | 0 | 0 |
| DNA binding, metabolism, replication and repair | 5,3 | 2,3 | 7,6 | 3,3 | 2,4 | 9,8 |
| Energy | 2,6 | 2,6 | 2,7 | 28,9 | 28,9 | 28,9 |
| Histone | 0 | 0 | 0 | 2,9 | 2,4 | 3,4 |
| Immune defence, xenobiotic metabolism, stress response | 3,3 | 2,3 | 15,9 | 4,3 | 2,3 | 50,5 |
| Kinase | 4,7 | 4,7 | 4,7 | 2,8 | 2,5 | 5,4 |
| Lipid, phospholipid, fatty acid metabolism and fatty acid beta oxidation | 3,9 | 2,3 | 76,8 | 2,8 | 2,3 | 8,3 |
| Membrane | 2,8 | 2,3 | 17,9 | 3,6 | 2,5 | 5,2 |
| Mitochondrial proteins and mitochondrial ribosomal proteins | 2,2 | 2,2 | 2,2 | 2,6 | 2,6 | 2,6 |
| Motor proteins and muscle | 3,5 | 2,2 | 14,1 | 2,7 | 2,3 | 9,4 |
| Neurogenesis | 3,7 | 2,2 | 32,6 | 2,8 | 2,3 | 4,8 |
| Nuclear ribosomal protein | 2,4 | 2,2 | 3,4 | 3,5 | 3,5 | 3,5 |
| Odorant binding proteins and receptors | 9,3 | 2,2 | 67,7 | 3,9 | 2,6 | 4,6 |
| Pathways and hormones | 3,6 | 2,2 | 78,3 | 2,9 | 2,2 | 13,7 |
| Protein metabolism, processing, stability, maitenance and repair | 3,1 | 2,3 | 7,7 | 3,4 | 2,3 | 46,8 |
| Receptor activity | 7,1 | 7,1 | 7,1 | 3,0 | 3,0 | 3,0 |
| Signaling | 2,7 | 2,6 | 3,4 | 2,7 | 2,3 | 5,7 |
| TCA | 0 | 0 | 0 | 10,5 | 2,8 | 18,1 |
| Transcription | 2,5 | 2,0 | 4,9 | 2,8 | 2,2 | 16,8 |
| Transferase activity | 3,2 | 2,3 | 5,9 | 4,2 | 2,4 | 24,4 |
| Translation | 3,4 | 3,2 | 3,7 | 3,7 | 3,7 | 3,7 |
| Transport | 3,5 | 2,2 | 19,4 | 3,1 | 2,2 | 22,5 |
| Unknown function | 3,3 | 2,2 | 250,5 | 3,1 | 2,2 | 36,4 |

| Supplementary table 11 | Foragers vs *vg*knockdowns | | | *vg*knockdowns vs foragers | | |
| --- | --- | --- | --- | --- | --- | --- |
| GO annotation | Median | Mina | Maxa | Median | Mina | Maxa |
| Autophagy, apoptosis and cell death | 4,6 | 4,6 | 4,6 | 3,8 | 3,8 | 3,8 |
| Behaviour, sensory, learning and memory | 3,4 | 2,9 | 126,8 | 3,4 | 2,9 | 4,6 |
| Binding | 3,0 | 2,9 | 4,4 | 3,1 | 2,8 | 3,5 |
| Carbohydrate metabolic process | 13,5 | 5,0 | 24,6 | 10,0 | 10,0 | 10,0 |
| Cell growth and development | 3,6 | 2,7 | 6,1 | 3,6 | 3,0 | 4,2 |
| Chaperone | 0 | 0 | 0 | 0 | 0 | 0 |
| Chromosome maitenance | 2,2 | 2,2 | 2,2 | 0 | 0 | 0 |
| Cuticular proteins, chitinase and chitin metabolic process | 12,6 | 2,9 | 52,4 | 3,2 | 3,2 | 3,2 |
| Determination of adult life span | 3,9 | 3,9 | 3,9 | 3,2 | 3,2 | 3,2 |
| DNA binding, metabolism, replication and repair | 3,3 | 3,2 | 9,6 | 3,5 | 3,5 | 3,5 |
| Energy | 0 | 0 | 0 | 4,0 | 4,0 | 4,0 |
| Histone | 0 | 0 | 0 | 4,2 | 4,2 | 4,2 |
| Immune defence, xenobiotic metabolism, stress response | 4,1 | 2,8 | 15,3 | 4,6 | 3,0 | 6,0 |
| Kinase | 5,7 | 5,6 | 5,8 | 3,3 | 2,8 | 4,0 |
| Lipid, phospholipid, fatty acid metabolism and fatty acid beta oxidation | 4,2 | 2,9 | 5,2 | 0 | 0 | 0 |
| Membrane | 8,8 | 7,3 | 10,4 | 3,5 | 3,0 | 4,1 |
| Mitochondrial proteins and mitochondrial ribosomal proteins | 0 | 0 | 0 | 4,6 | 3,5 | 5,8 |
| Motor proteins and muscle | 2,7 | 2,1 | 3,2 | 0 | 0 | 0 |
| Neurogenesis | 5,1 | 2,9 | 20,7 | 5,6 | 5,6 | 5,6 |
| Nuclear ribosomal protein | 0 | 0 | 0 | 3,4 | 3,4 | 3,4 |
| Odorant binding proteins and receptors | 0 | 0 | 0 | 0 | 0 | 0 |
| Pathways and hormones | 3,5 | 2,9 | 4,7 | 3,4 | 2,3 | 7,2 |
| Protein metabolism, processing, stability, maitenance and repair | 9,7 | 2,8 | 56,5 | 3,2 | 2,7 | 4,5 |
| Receptor activity | 3,7 | 3,7 | 3,7 | 2,9 | 2,6 | 3,2 |
| Signaling | 2,8 | 2,6 | 17,7 | 0 | 0 | 0 |
| TCA | 0 | 0 | 0 | 2,8 | 2,8 | 2,8 |
| Transcription | 3,8 | 2,8 | 7,0 | 3,4 | 3,1 | 4,6 |
| Transferase activity | 4,7 | 3,2 | 18,4 | 3,6 | 3,0 | 5,0 |
| Translation | 3,5 | 3,4 | 3,6 | 3,7 | 3,0 | 3,9 |
| Transport | 5,2 | 3,1 | 22,2 | 5,1 | 5,1 | 5,1 |
| Unknown function | 4,1 | 2,4 | 48,1 | 3,3 | 2,9 | 5,9 |

| Supplementary table 12 | Foragers vs methoprene | | | Methoprene vs foragers | | |
| --- | --- | --- | --- | --- | --- | --- |
| GO annotation | Median | Mina | Maxa | Median | Mina | Maxa |
| Autophagy, apoptosis and cell death | 3,3 | 3,3 | 3,3 | 3,3 | 2,8 | 3,7 |
| Behaviour, sensory, learning and memory | 11,2 | 2,6 | 121,3 | 3,0 | 2,7 | 3,1 |
| Binding | 3,2 | 2,6 | 10,0 | 2,8 | 2,6 | 4,7 |
| Carbohydrate metabolic process | 5,8 | 3,1 | 14,5 | 3,4 | 2,3 | 3,7 |
| Cell growth and development | 4,2 | 2,6 | 7,3 | 3,1 | 3,0 | 3,6 |
| Chaperone | 2,8 | 2,7 | 3,5 | 7,4 | 2,9 | 24,0 |
| Chromosome maitenance | 3,9 | 3,9 | 3,9 | 0 | 0 | 0 |
| Cuticular proteins, chitinase and chitin metabolic process | 8,9 | 2,7 | 54,6 | 3,3 | 3,0 | 3,6 |
| Determination of adult life span | 0 | 0 | 0 | 3,6 | 2,7 | 4,5 |
| DNA binding, metabolism, replication and repair | 2,8 | 2,6 | 9,1 | 3,1 | 3,1 | 3,1 |
| Energy | 0 | 0 | 0 | 0 | 0 | 0 |
| Histone | 0 | 0 | 0 | 4,0 | 4,0 | 4,0 |
| Immune defence, xenobiotic metabolism, stress response | 4,1 | 3,1 | 18,5 | 3,4 | 2,8 | 4,0 |
| Kinase | 4,5 | 4,3 | 4,6 | 3,4 | 3,0 | 6,4 |
| Lipid, phospholipid, fatty acid metabolism and fatty acid beta oxidation | 4,7 | 3,8 | 11,6 | 2,7 | 2,7 | 2,7 |
| Membrane | 5,1 | 4,0 | 6,2 | 3,9 | 3,2 | 4,6 |
| Mitochondrial proteins and mitochondrial ribosomal proteins | 0,0 | 0,0 | 0,0 | 3,0 | 2,9 | 3,1 |
| Motor proteins and muscle | 3,3 | 3,2 | 3,4 | 3,0 | 2,8 | 5,0 |
| Neurogenesis | 10,0 | 3,2 | 18,3 | 3,1 | 2,8 | 9,8 |
| Nuclear ribosomal protein | 0 | 0 | 0 | 0 | 0 | 0 |
| Odorant binding proteins and receptors | 0 | 0 | 0 | 3,3 | 2,7 | 3,9 |
| Pathways and hormones | 3,7 | 3,0 | 4,8 | 3,2 | 2,9 | 4,8 |
| Protein metabolism, processing, stability, maitenance and repair | 8,8 | 2,9 | 67,1 | 3,5 | 3,0 | 4,4 |
| Receptor activity | 0 | 0 | 0 | 3,5 | 2,9 | 4,0 |
| Signaling | 7,1 | 5,0 | 12,2 | 0 | 0 | 0 |
| TCA | 0 | 0 | 0 | 11,5 | 11,5 | 11,5 |
| Transcription | 3,3 | 2,7 | 6,9 | 2,9 | 2,4 | 4,4 |
| Transferase activity | 6,9 | 3,9 | 10,1 | 3,5 | 2,8 | 7,7 |
| Translation | 3,3 | 3,3 | 3,3 | 0 | 0 | 0 |
| Transport | 4,3 | 2,6 | 18,6 | 3,3 | 2,8 | 5,7 |
| Unknown function | 5,1 | 2,6 | 65,5 | 3,0 | 1,9 | 5,7 |

| Supplementary table 13 | Foragers vs reversion | | | Reversion vs foragers | | |
| --- | --- | --- | --- | --- | --- | --- |
| GO annotation | Median | Mina | Maxa | Median | Mina | Maxa |
| Autophagy, apoptosis and cell death | 0 | 0 | 0 | 0 | 0 | 0 |
| Behaviour, sensory, learning and memory | 27,0 | 11,2 | 42,9 | 5,2 | 5,2 | 5,2 |
| Binding | 0 | 0 | 0 | 2,8 | 2,6 | 3,0 |
| Carbohydrate metabolic process | 10,8 | 8,6 | 12,9 | 2,8 | 2,5 | 3,4 |
| Cell growth and development | 0 | 0 | 0 | 3,2 | 3,0 | 3,5 |
| Chaperone | 0 | 0 | 0 | 0 | 0 | 0 |
| Chromosome maitenance | 0 | 0 | 0 | 0 | 0 | 0 |
| Cuticular proteins, chitinase and chitin metabolic process | 33,9 | 14,9 | 52,8 | 2,9 | 2,9 | 2,9 |
| Determination of adult life span | 0 | 0 | 0 | 2,9 | 2,7 | 2,7 |
| DNA binding, metabolism, replication and repair | 9,6 | 9,6 | 9,6 | 2,8 | 2,5 | 3,0 |
| Energy | 0 | 0 | 0 | 0 | 0 | 0 |
| Histone | 0 | 0 | 0 | 2,7 | 2,7 | 2,7 |
| Immune defence, xenobiotic metabolism, stress response | 9,9 | 9,9 | 9,9 | 2,8 | 2,5 | 3,3 |
| Kinase | 0 | 0 | 0 | 4,1 | 2,8 | 5,3 |
| Lipid, phospholipid, fatty acid metabolism and fatty acid beta oxidation | 13,6 | 13,6 | 13,6 | 3,4 | 3,4 | 3,4 |
| Membrane | 0 | 0 | 0 | 0 | 0 | 0 |
| Mitochondrial proteins and mitochondrial ribosomal proteins | 0 | 0 | 0 | 3,1 | 2,8 | 3,7 |
| Motor proteins and muscle | 0 | 0 | 0 | 2,5 | 2,5 | 2,5 |
| Neurogenesis | 15,2 | 11,3 | 19,1 | 3,6 | 2,4 | 5,7 |
| Nuclear ribosomal protein | 0 | 0 | 0 | 3,5 | 3,5 | 3,5 |
| Odorant binding proteins and receptors | 0 | 0 | 0 | 4,5 | 3,3 | 5,0 |
| Pathways and hormones | 0 | 0 | 0 | 2,6 | 2,5 | 3,6 |
| Protein metabolism, processing, stability, maitenance and repair | 16,4 | 9,0 | 27,9 | 2,8 | 2,5 | 4,0 |
| Receptor activity | 0 | 0 | 0 | 3,6 | 2,1 | 3,1 |
| Signaling | 0 | 0 | 0 | 2,9 | 3,6 | 3,6 |
| TCA | 0 | 0 | 0 | 3,2 | 2,4 | 3,9 |
| Transcription | 0 | 0 | 0 | 3,2 | 2,8 | 3,9 |
| Transferase activity | 11,5 | 11,5 | 11,5 | 2,9 | 2,2 | 6,0 |
| Translation | 0 | 0 | 0 | 2,7 | 2,7 | 2,7 |
| Transport | 12,9 | 9,2 | 16,6 | 3,1 | 2,6 | 3,1 |
| Unknown function | 20,8 | 8,5 | 33,2 | 2,8 | 2,5 | 5,9 |
