# Supplementary material for: Somatic Maintenance Resources in the Honeybee Worker Fat Body Are Distributed to Withstand the Most Life-Threatening Challenges at Each Life Stage
Source: PLoS One. 2013 Aug 5;8(8):e69870. doi: 10.1371/journal.pone.0069870 (PMC3734224; doi:10.1371/journal.pone.0069870)
Supplement: Table S4 — Primer design for qPCR analysis. (DOCX) [file pone.0069870.s006.docx]

*Supplementary table 4: Primers for qPCR.*
